# An Integrated Proteomics and Bioinformatics Approach Reveals the Anti-inflammatory Mechanism of Carnosic Acid

**DOI:** 10.3389/fphar.2018.00370

**Published:** 2018-04-16

**Authors:** Li-Chao Wang, Wen-Hui Wei, Xiao-Wen Zhang, Dan Liu, Ke-Wu Zeng, Peng-Fei Tu

**Affiliations:** ^1^State Key Laboratory of Natural Medicines, China Pharmaceutical University, Nanjing, China; ^2^State Key Laboratory of Natural and Biomimetic Drugs, School of Pharmaceutical Sciences, Peking University, Beijing, China; ^3^Proteomics Laboratory, Medical and Healthy Analytical Center, Peking University Health Science Center, Beijing, China

**Keywords:** carnosic acid, anti-inflammatory, proteomics, MAPK pathway, IKKβ/IκB-α/NF-κB pathway, FoxO1/3 pathway

## Abstract

Drastic macrophages activation triggered by exogenous infection or endogenous stresses is thought to be implicated in the pathogenesis of various inflammatory diseases. Carnosic acid (CA), a natural phenolic diterpene extracted from *Salvia officinalis* plant, has been reported to possess anti-inflammatory activity. However, its role in macrophages activation as well as potential molecular mechanism is largely unexplored. In the current study, we sought to elucidate the anti-inflammatory property of CA using an integrated approach based on unbiased proteomics and bioinformatics analysis. CA significantly inhibited the robust increase of nitric oxide and TNF-α, downregulated COX2 protein expression, and lowered the transcriptional level of inflammatory genes including *Nos2*, *Tnfα*, *Cox*2, and *Mcp*1 in LPS-stimulated RAW264.7 cells, a murine model of peritoneal macrophage cell line. The LC-MS/MS-based shotgun proteomics analysis showed CA negatively regulated 217 LPS-elicited proteins which were involved in multiple inflammatory processes including MAPK, nuclear factor (NF)-κB, and FoxO signaling pathways. A further molecular biology analysis revealed that CA effectually inactivated IKKβ/IκB-α/NF-κB, ERK/JNK/p38 MAPKs, and FoxO1/3 signaling pathways. Collectively, our findings demonstrated the role of CA in regulating inflammation response and provide some insights into the proteomics-guided pharmacological mechanism study of natural products.

## Introduction

Macrophages, the innate immune cells, play a predominant role in tissue inflammation response for eliminating invading pathogens (Gao et al., 2017; Wu et al., 2017). Nevertheless, persistent or uncontrolled macrophage activation usually leads to the aberrant release of inflammatory cytokines and tissue damage (Gao et al., 2017). In recent decades, considerable investigations have shown that macrophage activation and subsequent inflammatory response contribute to the pathogenesis of a variety of serious diseases, including, but not limited to the tumor, cardiovascular disease, neurodegenerative disorder, obesity, and diabetes (Ku et al., 2016; Mondal et al., 2016; Reyes-Farias et al., 2016; Liou et al., 2017).

Plant-derived natural products are gaining worldwide attention for the treatment of bacterial infection and inflammatory diseases. *Salvia officinalis* L. (sage), a famous edible and medical herb, has been used as a dietary supplement and disinfector for thousands of years in Europe, China, and Japan (Scheler et al., 2016; Jiang et al., 2017). Carnosic acid (CA) is a natural phenolic diterpene originally isolated from *S. officinalis* plant and has been identified as one of the principal active components. To date, CA has been well proved as a potent antioxidant that is applied in food, health, and cosmetics industries (Birtić et al., 2015). Recently, its potential anti-inflammatory activity has attracted great attention. A number of investigations have demonstrated the anti-inflammatory role of CA in different *in vivo* and *in vitro* models, such as carrageenan-induced mouse hyperalgesia, collagen-induced arthritis, RANKL-induced osteoclastogenesis, and dextran sulfate sodium-induced acute colitis (Maione et al., 2017; Thummuri et al., 2017; Xia et al., 2017; Yang et al., 2017). Efforts directed at mechanisms of CA-mediated anti-inflammation found that CA reduced reactive oxygen species (ROS) production, up-regulated Keap1/Nrf2 pathway, attenuated nuclear factor (NF)-κB and p38/ERK1/2 MAP kinase signaling activation, and induced dephosphorylation of the forkhead box protein O3a (FoxO3a) (Schwager et al., 2016; Shibata et al., 2016; Thummuri et al., 2017; Xia et al., 2017; Yang et al., 2017).

Although such progress has made in profiling the potential of CA in inflammatory disease, the precise mechanism underlying has not been clearly characterized. Proteomics provides an effective strategy to investigate the entire spectrum of protein changes at a specific physiological condition (Kobeissy and Stevens, 1997). Proteomics emerge as a puissant tool to map the signal transduction pathways in inflammatory response and predict molecular mechanisms for anti-inflammatory remedies. In the present study, we performed an integrated approach based on label-free quantitative proteomics and bioinformatics analysis to uncover the mechanisms involved in the anti-inflammatory effects of CA. And our results reveal that CA confers its disruption on macrophages activation by inhibiting multiple signaling pathways including NF-κB, MAP kinase, and FoxO1/3 pathway, and subsequently effectively suppresses LPS-induced production of various inflammatory cytokines. These findings demonstrate the anti-inflammatory effect of CA and also provide a novel insight into the molecular pathway through which CA maintains macrophage homeostasis and inhibits inflammatory responses.

## Materials and Methods

### Chemicals and Reagents

Carnosic acid (C_20_H_28_O_4_; molecular weight 332.4400) was obtained from Baoji Herbest Bio-Tech Co., Ltd. (Shanxi, China) and the purity (above 98%) was affirmed by HPLC and MS data. Lipopolysaccharide (LPS) from *Escherichia coli* O55:B5 was from Sigma-Aldrich (St. Louis, MO, United States). MG132 was from Beyotime Biotechnology (Jiangsu, China). Antibodies against COX2 (12282), GAPDH (3683), p-IKKα/β (2697), IKKα (11930), IKKβ (8943), p-IκB-α (2859), IκB-α (4814), p-NF-κB p65 (3033), NF-κB p65 (8242), p-ERK1/2 MAPK (4370), p-SAPK/JNK (4668), SAPK/JNK (9252), p-p38 MAPK (4511), p38 MAPK (8690), Histone H3 (4499), α-Tubulin (9099), rabbit IgG (7074), and mouse IgG (7076) were purchased from Cell Signaling Technology (Beverly, MA, United States). Antibody against ERK1/2 MAPK (16443-1-AP) and ERK4 (26102-1-AP) was bought from Proteintech Group (Chicago, IL, United States). Antibodies against FKHR/FoxO1 (BS5518), FoxO3 (BS3574), ERK3 (BS2662), and p-ERK3/4 (BS6377) were from Bioworld Technology (St. Louis Park, MN, United States).

### Cell Culture

Murine RAW264.7 macrophage cell line was purchased from Peking Union Medical College, Cell Bank, China. Cells were routinely maintained in high glucose Dulbecco’s Modified Eagle Medium (DMEM) containing 10% heat-inactivated fetal bovine serum (FBS, PAN-Biotech, Aidenbach, Germany), 100 U/ml penicillin, and 100 μg/ml streptomycin at 37°C in a 5% CO_2_ humidified incubator.

### Cell Viability Assay

RAW264.7 cells were cultured in DMEM containing various concentrations of CA (0, 2.5, 5, 10, and 20 μM) with or without 1 μg/ml of LPS for 24 h. Cell viability was then determined by adding 3-(4, 5-dimethyl thiazol-2-yl)-2, 5-diphenyl tetrazolium bromide (MTT) solution (Sigma-Aldrich, St. Louis, MO, United States) and incubating cells at 37°C for another 4 h. The formazan crystal products were dissolved in DMSO and the absorbance was measured at 570 nm.

### Nitrite Oxide (NO) Production Assay

After treatment with different concentrations of CA (0, 2.5, 5, 10, and 20 μM) in the presence or absence of LPS (1 μg/ml) for 24 h, the cell culture supernatants were collected and the accumulation of NO in the culture medium was evaluated by the Griess method with Nitric oxide (NO) assay kit (Jiancheng Bioengineering Institute, Nanjing, Jiangsu, China) following the manufacturer’s instructions.

### ELISA for TNF-α

RAW264.7 cells were treated with 1 μg/ml of LPS containing CA (0, 2.5, 5, 10, and 20 μM) for 4 h. Culture supernatants were then collected and centrifuged prior to the determination of TNF-α level with a commercially available ELISA kit (ExCell Bio Company, Shanghai, China). Detailed manipulation process was performed in accordance with the protocol of manufacturer.

### Western Blot Analysis

After treatment, cells were collected and washed by PBS for twice. Cells were homogenized with ice-cold NP-40 buffer for 30 min to provide the whole cell proteins. Nuclear and cytoplasmic proteins were prepared using the Nuclear and Cytoplasmic Protein Extraction Kit (Beyotime) accordance to the manufacturer’s protocol. The concentrations of total proteins, nuclear proteins, and cytoplasmic proteins were determined using enhanced BCA protein assay reagent (TransGen Biotech, Beijing, China), respectively. Equal amounts of proteins extracts were separated on 8–12% SDS–PAGE gels and subsequently transferred onto polyvinylidene fluoride membranes. Membranes were blocked in 5% skimmed milk solution and then probed with diluted primary antibodies (1:1000) overnight at 4°C. After incubating with HRP-conjugated anti-rabbit or anti-mouse IgG secondary antibody, protein bands were developed with enhanced chemiluminescence (ECL) substrate and visualized by Tanon 5200 Imaging Analysis System (Tanon, Shanghai, China). Relative protein levels were performed by densitometry analysis using Image J software.

### RNA Extraction and Real-Time PCR Analysis

Cells were treated with 1 μg/ml of LPS in the absence or the presence of CA (5, 10, and 20 μM) for 6 h. Then, cells were harvested and the total RNA was extracted using EasyPure RNA Kit (TransGen Biotech, Beijing, China). The mRNA was reverse transcribed into cDNA by TransScript First-Strand cDNA Synthesis SuperMix (TransGen Biotech, Beijing, China). Quantitative real-time PCR (qRT-PCR) was then carried out as 40 cycles of 95°C for 30 s, 58°C for 30 s, and 72°C for 30 s on Agilent Technologies Stratagene Mx3005P. The sequences of the PCR primers used in this study are listed in Supplementary Table 1. The threshold cycle (CT) values were provided at the end of PCR. The relative transcriptional level of target genes normalized to GAPDH was calculated by the comparative 2^-ΔΔ^ ^CT^ method (Liao et al., 2017).

### Immunofluorescence Assay

Cells were seeded on glass over slips in 24-well plates. After overnight incubation, the culture medium was removed and replaced with 1 μg/ml of LPS containing CA (5, 10, and 20 μM) or vehicle. At 1 h after incubation, cells were fixed with 4% paraformaldehyde followed by permeabilizing in 0.5% Triton X100 and blocking in 5% BSA. Afterward, the coverslips were sequentially incubated with diluted primary antibody against NF-κB p65 (1: 400) overnight at 4°C, Alexa Fluor 594-labeled secondary antibody for 1 h at room temperature, and DAPI (50 μg/ml) for 20 min. Image acquisition was achieved using Olympus IX73 fluorescence microscope (Tokyo, Japan).

### Protein Identification by Nano LC-MS/MS

#### Samples Preparation

The cells cultured with 1 μg/ml of LPS or CA-contained LPS for 24 h were harvested and washed by ice-cold PBS. The total proteins were isolated using ice-cold NP-40 buffer and quantified using enhanced BCA method. Equal amounts of extracts were resolved by SDS–PAGE and stained by a fast silver stain kit (Beyotime). Whole-protein bands on SDS–PAGE were excised for each sample and then digested with trypsin. The resultant tryptic peptides were filtered through a 0.22-μm micro-pore membrane to provide MS samples.

#### Mass Spectrometry Analysis

Extracted peptide samples were analyzed by nano-liquid chromatography coupled with hybrid linear ion trap-Orbitrap mass spectrometer (nanoLC-LTQ-Orbitrap MS/MS) method. The chromatographic separation was performed on an EASY -nLC II system (Thermo Fisher Scientific, United States) equipped with a RP-C18AQ column (100 μm id × 15 cm, Michrom Bioresources, United States). The mobile phase consisted of 0.1% formic acid in water (A) and 0.1% formic acid in acetonitrile (B) was delivered at a constant flow rate of 300 nl/min. The following gradient elution program was adopted: 2–40% B for 70 min, 40–95% B for 5 min, and 95% B for 10 min. The injected sample volume in HPLC and MS was set at 1 and 10 μl, respectively.

Data acquisition was conducted using a data-dependent strategy on a high resolution LTQ-Orbitrap Velos Pro hybrid mass spectrometer (Thermo Fisher Scientific, United States). The Orbitrap mass spectrometer was equipped with a nano-electrospray ion source with an ion spray voltage of 1.8 kV and the Orbitrap analyzer with a resolution of 60,000 (FWHM). Full scan MS spectrum was recorded across the range *m*/*z* 350–2000 in the positive mode, of which the top 15 most abundant ion signals were selected as precursors for further LTQ-MS/MS scans. Collision energy for CID was set at 25 eV. The raw MS/MS data were then processed and searched for the protein identification in Thermo Proteome Discoverer (v.1.4.1.14) software.

### Bioinformatics Analysis

The Gene Ontology (GO) protein classification analysis according to cellular component (CC), molecular function (MF), and biological process (BP) was carried out using Database for Annotation, Visualization and Integrated Discovery (DAVID^1^) and Protein Analysis Through Evolutionary Relationships database (PANTHER^2^). The signaling pathway enrichment analysis was performed using the Kyoto Encyclopedia of Genes and Genomes (KEGG), REACTOME pathway, and Wiki pathway database from ClueGO program, a plug-in Cytoscape software (v.3.5.1).

### Statistical Analysis

All data were expressed as mean ± standard error of the mean (SEM). Statistical analyses were performed using GraphPad Prism 6.0 software. Mean values were compared by one-way analysis of variance (ANOVA) with Bonferroni’s *post hoc* test. *P* < 0.05 was considered as statistically significant.

## Results

### CA Attenuates the Secretion of Various Inflammatory Mediators in LPS-Challenged RAW264.7 Cells

**FIGURE 1:**
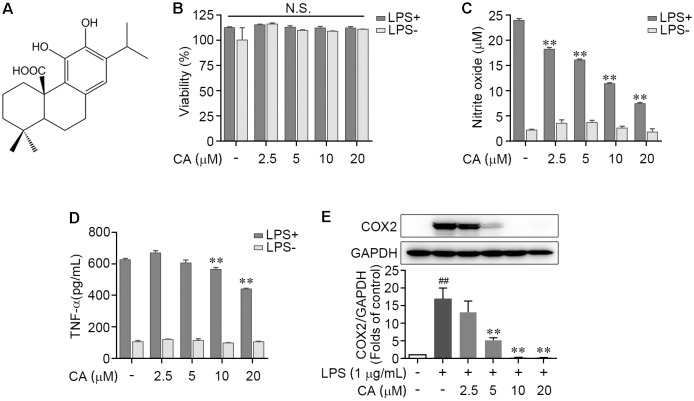
Carnosic acid down-regulated the levels of pro-inflammation mediators in LPS-induced RAW264.7 cells. **(A)** Chemical structure of carnosic acid (CA). **(B,C)** RAW264.7 cells were treated with various concentration of CA (2.5, 5, 10, and 20 μM) in the absence or presence of LPS (1 μg/ml) for 24 h. Then the cell viability **(B)** and nitric oxide production **(C)** were determined by MTT and Griess methods, respectively. **(D)** RAW274.7 cells were treated with CA and LPS as in **(B,C)** for 4 h, then the TNF-α level was detected by ELISA. **(E)** RAW264.7 cells were treated with 1 μg/ml of LPS containing CA (2.5, 5, 10, and 20 μM) or not for 24 h. The protein expression of COX2 was detected by western blot assay. Data are expressed as mean ± SEM from three individual experiments. ^∗∗^*P* < 0.001 vs. LPS group; ^##^*P* < 0.01 vs. control group; N.S., not significant by ANOVA with Bonferroni’s *post hoc* test.

We first tested the potential cytotoxicity of CA on LPS-stimulated RAW264.7 cells by utilizing MTT method. The dose of CA was set as 2.5, 5, 10, and 20 μM according to prior studies (Park and Mun, 2014; Thummuri et al., 2017; Xia et al., 2017). Results showed that there was no significant difference between control and CA treatments at the indicated concentrations for 24 h on cell viability (**Figure 1B**). Furthermore, 1 μg/ml of LPS, alone or co-treatment with different concentrations of CA, did not distinctly affect the cell survival (**Figure 1B**). Thus, CA at the concentrations of 2.5, 5, 10, and 20 μM was adopted to analyze its role in LPS-evoked inflammatory response. As shown in **Figures 1C,D**, treatment with CA itself exhibited faint changes on the NO and TNF-α levels. LPS stimulation led to a robust increase of these two inflammatory agents, which were effectually blocked by CA in a dose-dependent manner (**Figures 1C,D**). Similarly, CA potently inhibited the LPS-elicited overexpression of COX2 protein in RAW264.7 cells (**Figure 1E**). Taken together, these findings demonstrated that LPS-caused inflammatory response in RAW264.7 cells was efficiently prevented by CA treatment.

### CA Prevents Inflammatory Genes Expression in LPS-Activated RAW264.7 Cells

**FIGURE 2:**
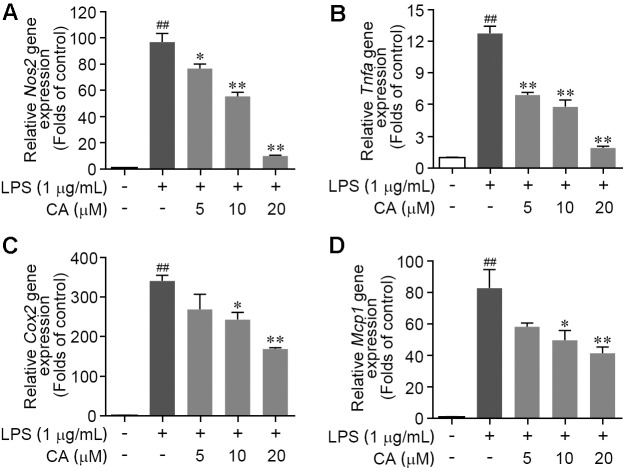
Carnosic acid down-regulated the levels of pro-inflammation gene expression in LPS-stimulated RAW264.7 cells. **(A–D)** Cells were treated with LPS (1 μg/ml) for 6 h with or without CA (5, 10, and 20 μM). The relative mRNA expressions of *Nos2* **(A)**, *Tnfα* **(B)**, *Cox2* **(C)**, and *Mcp1* **(D)** were detected by real-time PCR analysis, respectively. Data are expressed as mean ± SEM (*n* = 3). ^∗^*P* < 0.05, ^∗∗^*P* < 0.01 vs. LPS group; ^##^*P* < 0.01 vs. control group by ANOVA with Bonferroni’s *post hoc* test.

To further validate the efficiency of CA on LPS-mediated acute inflammatory response in RAW264.7 cells, the gene expressions of pro-inflammatory cytokines including iNOS, TNF-α, and COX2 were assessed by RT-PCR method. As described in **Figures 2A–C**, LPS treatment significantly elevated the mRNA transcript levels of *Nos2*, *Tnfα*, and *Cox2*; these alterations were concentration-dependently reversed by CA treatment (5, 10, and 20 μM). In addition, we found that the LPS-induced gene expression increase of monocyte chemotactic protein (MCP-1), one of pivotal chemokines, was evidently restrained by CA at various concentrations (**Figure 2D**). These results manifested that CA functions as a versatile inhibitor against LPS-induced RAW264.7 macrophage activation and acute inflammation.

### Effects of CA on Proteomic Changes Induced by LPS in RAW264.7 Cells

**FIGURE 3:**
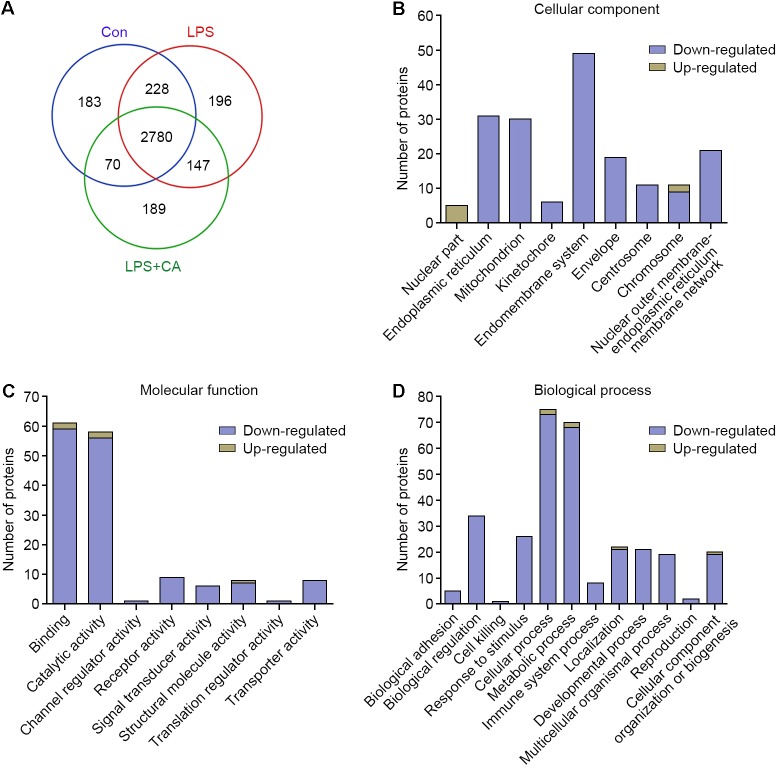
Outputs of proteomic analysis for CA-altered proteins. **(A)** Venn diagram showing numerical distribution of proteins identified in different RAW264.7 cell lysates by the nanoLC-LTQ-Orbitrap MS/MS approach. Cells were treated with vehicle (control group), LPS (1 μg/ml, LPS group), and LPS with 20 μM of CA (LPS+CA group) for 6 h, respectively. **(B–D)** Proteins significantly up-regulated and down-regulated by CA were classified into different cellular components **(B)**, molecular functions **(C)**, and BPs **(D)**.

Nano-LC-MS/MS system for quantitative proteomics was carried out to dissect the effect of CA on proteome profile. Protein identification of the RAW264.7 cell lysates against the Thermo Proteome Discoverer database showed a total of 3261, 3351, and 3186 proteins were in the control, LPS, and LPS+CA groups, respectively. The relationship of identified proteins in the three groups was described in a Venn diagram (**Figure 3A**).

The expression levels of the whole proteins in different groups were compared to give the differentially expressed protein candidates. In this study, protein with a more than fivefold increase or decrease was filtrated. Based on this criterion, 718 differentially expressed proteins were identified. Specifically, 428 proteins were up-regulated and 290 were down-regulated upon LPS treatment. Likewise, the 718 differentially expressed proteins were further analyzed between LPS and LPS+CA groups. Results showed that 217 (50.7%) up-regulated proteins and 9 (3.1%) down-regulated proteins in LPS group were markedly reversed in response to CA treatment (Supplementary Data 1).

To explore the impact of CA on proteomic changes, these proteins specifically responded to CA treatment were annotated and categorized according to GO CC, MF, and BP. As shown in **Figure 3B**, the major cellular localizations of these proteins are endomembrane system (49 proteins), endoplasmic reticulum (31 proteins), and mitochondrion (30 proteins). Functionally, they possess diversified activities, for instance, binding (59 proteins), catalytic activity (56 proteins), receptor activity (9 proteins), and signal transducer activity (6 proteins) (**Figure 3C**). Moreover, these proteins are involved in complicated BPs including cellular process (73 proteins), metabolic process (68 proteins), immune system process (8 proteins), and so on (**Figure 3D**). Therefore, these results indicated that CA principally negatively regulated the LPS-irritated proteins expressions and multiple BPs.

### Bioinformatics Analysis for the CA-Regulated Signaling Networks

**FIGURE 4:**
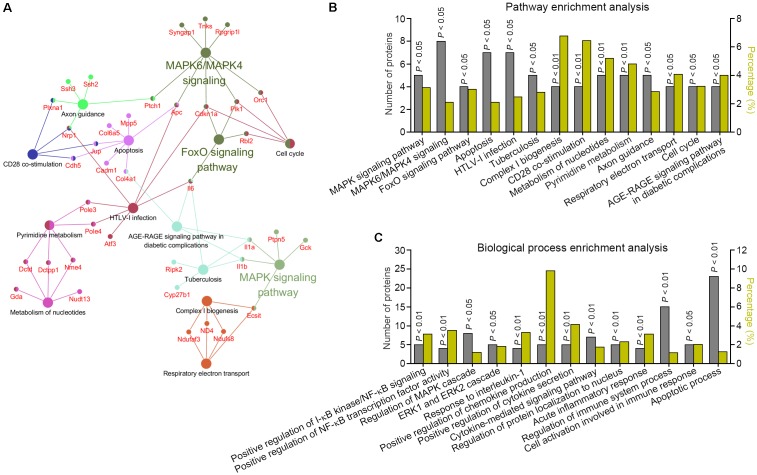
Bioinformatics analysis of the signaling networks negatively regulated by CA. **(A,B)** Pathway enrichment analysis for the differentially expressed proteins predicted the significantly canonical pathways targeted by CA in LPS-stimulated RAW264.7 cells. The gray columns plotting on the left *Y*-axis depict the number of identified proteins found in each pathway. The olive yellow columns plotting on the right *Y*-axis depict the percentage of identified proteins over the total proteins in that pathway. **(C)** BP enrichment analysis for differentially expressed proteins in CA-treated RAW264.7 cells.

To visualize CA-regulated pharmacological network, the 217 CA-negatively regulated proteins were examined for enrichment in KEGG pathway, REACTOME pathway, and Wiki pathway database from ClueGO plug-in. 125 proteins were functionally annotated in these selected ontologies. The global pathway network depicting GO term enrichment of *P* < 0.05 was mapped in **Figure 4A** and listed in **Figure 4B** and Supplementary Table 2. Three major enriched pathways were yielded, including MAPK pathway, MAPK6/MAPK4 pathway, and FoxO pathway. To confirm this, GO BP enrichment analysis related to the above functionally annotated proteins was utilized. Result showed that multiple inflammatory response-associated BPs were significantly enriched, including positive regulation of IκB kinase/NF-κB signaling, positive regulation of NF-κB transcription factor activity, regulation of MAPK cascade, ERK1 and ERK2 cascade, response to interleukin-1, positive regulation of chemokine production, positive regulation of cytokine secretion, cytokine-mediated signaling pathway, regulation of protein localization to nucleus, acute inflammatory response, regulation of immune system process, and cell activation involved in immune response (**Figure 4C** and Supplementary Table 3). Together, these findings suggested that CA could effectively extinguish multiple LPS-activated inflammatory pathways and BPs, and thereby exerted anti-inflammation activity.

### CA Down-Regulated MAP Kinase Pathway in LPS-Induced RAW264.7 Cells

**FIGURE 5:**
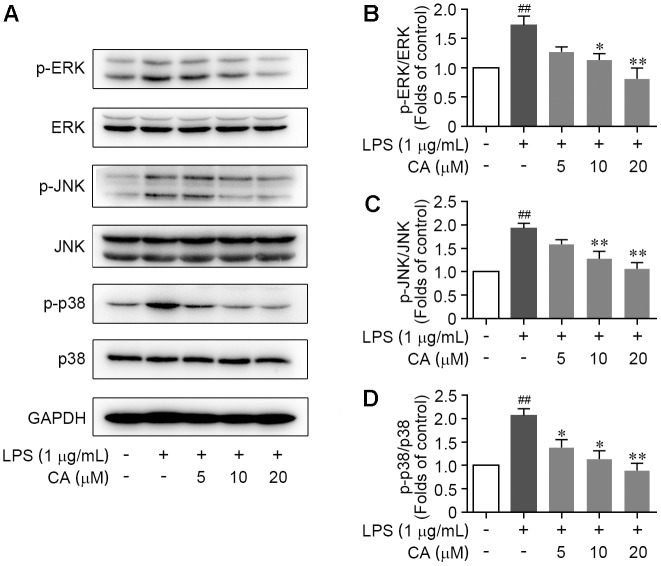
CA restrained the activation of ERK, JNK, and p38 MAPKs in LPS-challenged RAW264.7 cells. Cells were treated with LPS (1 μg/ml) with or without CA (5, 10, and 20 μM) for 1 h. **(A)** Phosphorylations of ERK, JNK, and p38 protein were determined by western blot assay. **(B–D)** Quantitative analysis for relative phosphorylation levels of ERK **(B)**, JNK **(C)**, and p38 MAPK **(D)** was performed by normalizing to the control group. Data are expressed as mean ± SEM from three individual experiments. ^∗^*P* < 0.05, ^∗∗^*P* < 0.01 vs. LPS group. ^##^*P* < 0.01 vs. control group by ANOVA with Bonferroni’s *post hoc* test.

As the initiation of mitogen-activated protein kinase (MAPK) signal transduction pathway has been widely manifested as a prominent feature in LPS-induced acute inflammation. We next examined the activations of ERK1/2, JNK, and p38, the hallmarks of canonical MAPK pathway by western blot to ascertain their roles for CA-mediated anti-inflammation function. As described in **Figures 5A–D**, MAPKs were highly activated upon LPS stimulation, as indicated by the significant induction of the phosphorylation of all three members including ERK, JNK, and p38, while such alterations were largely attenuated by CA treatment in a concentration-dependent fashion (**Figures 5A–D**). Furthermore, the changes of atypical MAPKs including ERK3 (MAPK6) and ERK4 (MAPK4) in RAW264.7 cells following LPS stimulation and CA treatment were validated by western blot method. Result showed that both LPS challenge and CA treatment have feeble effect on the activation of MAPK6 and MAPK4 (Supplementary Figures 1A–C). These above results illustrated that the extensive and intense down-regulation of CA on canonical MAPK pathway should attribute to its anti-inflammatory effect.

### CA Antagonized LPS-Induced IKKβ/IκB-α/NF-κB Pathway Activation

**FIGURE 6:**
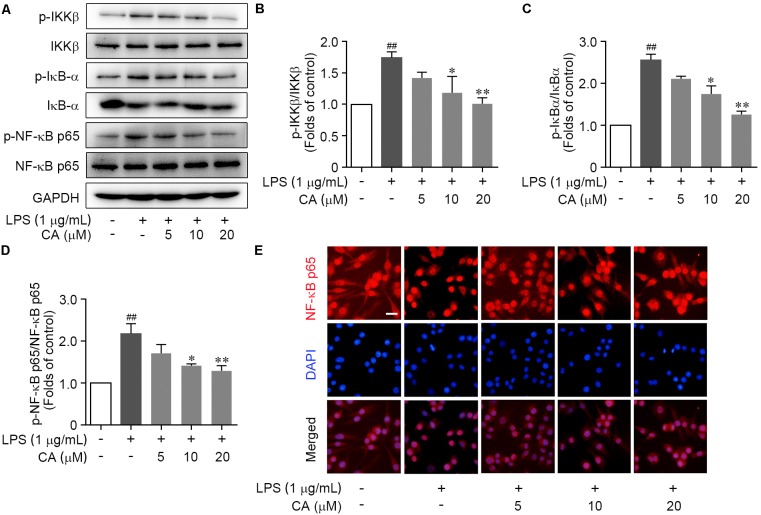
CA suppressed LPS-induced IKKβ/IκB-α/NF-κB signaling activation in RAW264.7 cells. Cells were treated with LPS (1 μg/ml) with or without CA (5, 10, and 20 μM) for 1 h. **(A)** Phosphorylation and total expressions of IKKβ, IκBα, and NF-κB p65 were determined by western blot assay. **(B–D)** Quantitative analysis for relative phosphorylation levels of IKKβ **(B)**, IκB-α **(C)**, and NF-κB p65 **(D)** was performed by normalizing to the control group. **(E)** The nuclear translocation of NF-κB p65 was detected by immunofluorescence assay. Representative images were displayed with NF-κB p65 (red) and nucleus (blue). Typical apoptotic neurons were labeled with white arrows. Scale bar = 40 μm. Data are expressed as mean ± SEM from three individual experiments. ^∗^*P* < 0.05, ^∗∗^*P* < 0.01 vs. LPS group. ^##^*P* < 0.01 vs. control group by ANOVA with Bonferroni’s *post hoc* test.

Bioinformatics analysis pointed out that CA widely regulated the canonical NF-κB pathway. To verify this, we investigated the change of key biomarkers of canonical NF-κB pathway in RAW264.7 cells following LPS stimulation by western blot. We found that the phosphorylation levels of IKKβ and IκB-α were markedly augmented due to the LPS stimulation, and meanwhile, IκB-α protein expression was markedly reduced (**Figures 6A–C**). However, CA treatment significantly decreased the phosphorylation levels of IKKβ and IκB-α, and blocked the IκB-α degradation (**Figures 6A–C**). Furthermore, we observed an elevated phosphorylation of NF-κB p65 subunit and its nuclear translocation from cytoplasm in LPS-induced RAW264.7 cells (**Figures 6A,D,E**). CA treatment significantly inactivated NF-κB p65 subunit, as indicated by decreasing its phosphorylation and subsequently suppressing the nuclear translocation (**Figures 6A,D,E**). Collectively, CA inhibited IKKβ/IκB-α/NF-κB pathway activation upon inflammatory situation.

### CA Suppressed FoxO1/3 Pathway in LPS-Induced RAW264.7 Cells

**FIGURE 7:**
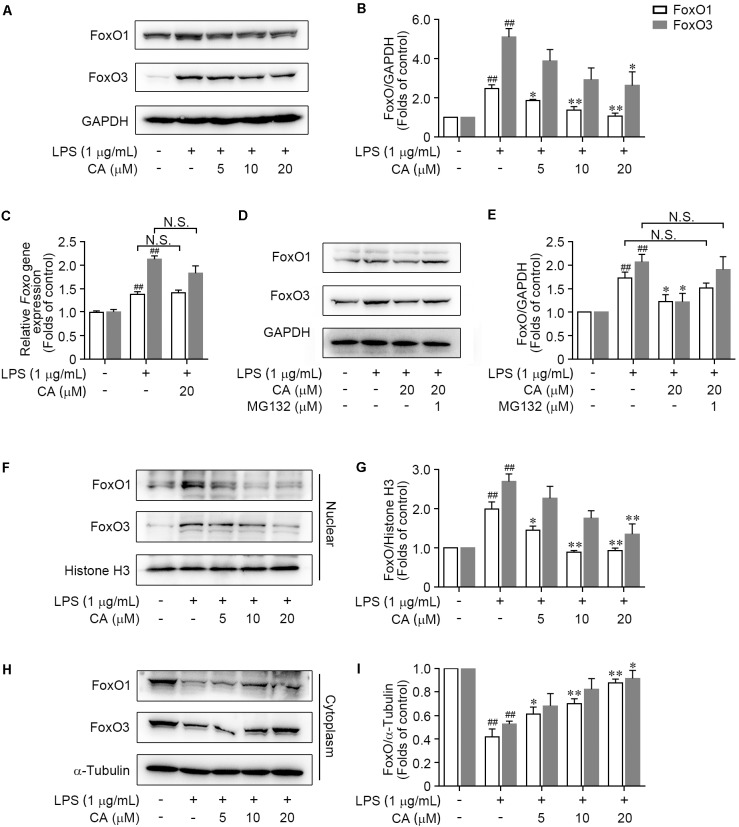
CA prevented FoxO1 and FoxO3 proteins translocating into the nucleus and promoted their degradation under LPS-challenged situation.chi-tang ho **(A,B)** RAW264.7 cells were treated with LPS (1 μg/ml) with or without CA (5, 10, and 20 μM) for 24 h. The total expressions of FoxO1 and FoxO3 were determined by western blot assay. **(C)** Cells treated as in **(A)** for 6 h were collected and subjected for real-time PCR experiment to detect the relative mRNA expressions of *FoxO1* and *FoxO3*. **(D,E)** The total expressions of FoxO1 and FoxO3 in RAW264.7 cells treated with LPS (1 μg/ml), CA (20 μM), and MG132 (1 μM) for 24 h were determined by western blot assay. **(F–I)** RAW264.7 cells were treated as in **(A)** for 1 h, then the nuclear and cytoplasmic expressions of FoxO1 and FoxO3 were detected by western blot assay. Histone H3 and α-tubulin were utilized as internal controls for nuclear and cytoplasmic proteins, respectively. Data are expressed as mean ± SEM from three individual experiments. ^∗^*P* < 0.05, ^∗∗^*P* < 0.01 vs. LPS group. ^##^*P* < 0.01 vs. control group by ANOVA with Bonferroni’s *post hoc* test.

As a family of transcription factors, FoxOs have been revealed to be triggered to transcribe target genes and thereby mediate the pro-inflammatory cytokines (Wątroba et al., 2012; Wang et al., 2015; Kashiwagi et al., 2017; Liu et al., 2017). As shown in **Figures 7A,B**, FoxO1 and FoxO3 protein expressions were significantly enhanced upon LPS stimulation. However, the overexpression of these two proteins was obviously restrained by CA in a concentration-dependent manner (**Figures 7A,B**). Interestingly, CA treatment did not show any effects on the gene expressions of *FoxO1* nor the *FoxO3* (**Figure 7C**). To further test whether CA inhibited FoxO1 and FoxO3 protein via proteasome proteolysis pathway, the proteasome inhibitor MG132 was further employed. As described in **Figures 7D,E**, treatment with MG132 could effectually reverse the reduction effect of CA on FoxO1 and FoxO3 protein levels, indicating CA could facilitate the degradations of FoxO1 and FoxO3 via ubiquitin-proteasome system.

We further explored the property of CA on the nuclear translocation of FoxO1/3, which is a key process for FoxO1/3-dependent biological function. Results showed that the nuclear FoxO1 expression was sharply increased in LPS-challenged RAW264.7 cells (**Figures 7F,G**), accompanied with the decrease of cytoplasmic FoxO1 level (**Figures 7H,I**). However, CA markedly blocked the nuclear translocation of FoxO1, indicated by diminishing the nuclear FoxO1 (**Figures 7F,G**) and elevating the cytoplasmic FoxO1 level (**Figures 7H,I**). In accordance with the regulation on FoxO1, CA treatment resulted in a concentration-dependent inhibition of nuclear translocation of FoxO3 (**Figures 7F–I**). Taken together, these findings indicated that CA abrogated the activation of FoxO pathway via suppressing nuclear translocation of FoxO1/3, and subsequently promoting the ubiquitin-dependent degradation of FoxO1/3.

## Discussion

The immortalized macrophage-like RAW 264.7 cell line is obtained from pristane-induced peritoneal macrophages transformed with Abelson murine leukemia virus (Maurya et al., 2013). RAW264.7 cells possess phenotypic resemblance to primary macrophages and could be cultured easily and massively, thereby have been widely applied as a simple and practical cell model for macrophage cellular physiology and inflammation response research (Mengoni et al., 2011; Chae et al., 2012; Maurya et al., 2013; Maione et al., 2017). In our present study, we investigated the anti-inflammation role of CA in LPS-induced RAW264.7 cells by examining the productions of various inflammatory cytokines. We found that CA significantly reduced the NO and TNF-α production and inhibited COX2 protein expression (Shibata et al., 2016; Thummuri et al., 2017). These beneficial effects were in consistent with previous studies (Oh et al., 2012; Xiang et al., 2013; Schwager et al., 2016). Several attempts have been made to reveal the mechanism by which CA affects inflammation response and showed that CA inhibited the activation of MAPK, NF-κB, and FoxO3a signaling under different inflammatory conditions (Schwager et al., 2016; Shibata et al., 2016; Thummuri et al., 2017; Xia et al., 2017). Whereas the global profile of CA-regulated inflammatory signaling networks remains to be elucidated.

Notably, the proteomics is increasingly applied for illuminating the molecular mechanisms of drugs. The proteomics aims to study the effect of drugs on the entire proteome changes and then find the relevant key signaling pathways through bioinformatics method. It is characterized by objectively and unbiasedly evaluating the pharmacological mechanism on a global level and thereby received much attention. So far, it has been successfully applied to the target identification and pharmacological mechanism studies for numerous well-known natural active molecules such as arsenic trioxide, ganoderic acid, and gambogic acid (Yue et al., 2008; Wang et al., 2009; Zhang et al., 2010). In this work, an integrated proteomics and bioinformatics approach was established to identify the CA-regulated proteins and their biological functions. Consequently, three critical inflammatory pathways were obtained and further confirmed as IKKβ/IκB-α/NF-κB, ERK/JNK/p38 MAPKs, and FoxO1/3 signaling pathway by using the molecular biological assays.

MAPK and NF-κB signaling cascades are two prominent canonical signaling pathways for driving inflammation response in almost all mammalian cells. According to the proteomic and bioinformatics analysis, we detected diverse CA-downregulated proteins that involved in MAPK and NF-κB signaling pathway in RAW264.7 cells. Correspondingly, the western blot experiment revealed that CA significantly inhibited the LPS-elicited phosphorylation of ERK1/2, JNK, and especially p38, indicative of its negative regulation on MAPK cascades, particularly on p38 MAPK pathway. Furthermore, CA antagonized LPS-induced canonical NF-κB pathway activation, including down-regulating the phosphorylation of IKKβ, IκB-α, and NF-κB p65 subunit and impeding IκB-α degradation. In light of these findings, the suppressive effect of CA on inflammatory genes expression may be a consequence relative with hampering the activation of MAPK and NF-κB pathway.

As two most well-understood FoxO family members, FoxO1 and FoxO3 transcription factors have been reported to regulate the generation of pro-inflammatory genes and relevant mediators such as IL6, TNF-α, and Mcp-1 (Savai et al., 2014; Kashiwagi et al., 2017; Liu et al., 2017). Moreover, inactivation of FoxO1 and especially FoxO3a showed benefits on mitochondria homeostasis and diminished ROS production in LPS-challenged macrophages (Priber et al., 2015). Hence, the FoxO1/3 may serve as feasible targets for inflammatory diseases. Based on this standpoint, FoxO1 and FoxO3 were further determined to ascertain the detailed mechanism whereby CA suppressed the FoxO pathway. In line with proteomic analysis, western blot and RT-PCR experiment corroborated the effectually inhibitory effects of CA on LPS-activated FoxO pathway, as evidenced by regulating the subcellular localization FoxO1 and FoxO3 protein and thereby lessening their protein levels via proteasome proteolysis pathway. In this regard, our work not only identified a beneficial role for CA in the regulation of FoxO pathway, but also indicated that direct pharmacological inactivation of FoxO1/3 might be effective in treating inflammatory diseases. Interestingly, JNK/p38 MAPKs have been reported to promote the nuclear localization and increase the transcriptional activity of FOXOs, among which FoxO1 serves as a coactivator of NF-κB (Wang et al., 2014). Moreover, the phosphorylation of IKKβ is revealed to engage in the ERK1/2-drived COX-2 gene expression (Chariot, 2009). Therefore, CA may exert anti-inflammatory effects through a mechanism involving crosstalk among the ERK/JNK/p38 MAPKs signaling, IKKβ/IκB-α/NF-κB axis, and FoxO1/3 pathway. In addition, it should be noted that there might be some differences between the RAW264.7 cell line and the primary macrophages. Although we have identified the CA-regulated inflammatory signaling networks in RAW264.7 cells, a further validation for these above crossed signaling pathways in primary macrophages and the identification of direct molecular target of CA is necessary in future studies.

In summary, the current study demonstrated the anti-inflammatory property of CA in a classical model of LPS-stimulated RAW264.7 macrophages and highlighted its related mechanism by an integrated approach of label-free quantitative proteomics and molecular biology analysis. CA, a natural compound, suppressed multiple LPS-activated pathways including IKKβ/IκB-α/NF-κB, ERK/JNK/p38 MAPKs, and FoxO1/3 signals to interrupt various inflammatory genes transcription and related cytokines production, and thereby exerts anti-inflammation function. These findings suggest the potential therapeutic targets for CA in the management of inflammatory diseases and offer insights into the proteomics-guided pharmacological mechanism study of natural products.

## Author Contributions

K-WZ and P-FT conceived and designed the research. L-CW performed most of the experiments. W-HW and X-WZ coordinated the experiments. DL performed the nano-LC-MS/MS analysis. K-WZ, P-FT, and L-CW wrote the manuscript.

## Conflict of Interest Statement

The authors declare that the research was conducted in the absence of any commercial or financial relationships that could be construed as a potential conflict of interest.

## Footnotes

1 http://david.abcc.ncifcrf.gov

2 http://pantherdb.org/
