# Supplementary material for: An Integrated Proteomics and Bioinformatics Approach Reveals the Anti-inflammatory Mechanism of Carnosic Acid
Source: Front Pharmacol. 2018 Apr 16;9:370. doi: 10.3389/fphar.2018.00370 (PMC5911474; doi:10.3389/fphar.2018.00370)
Supplement: Supplementary file 1 [file Table_1.docx]

**
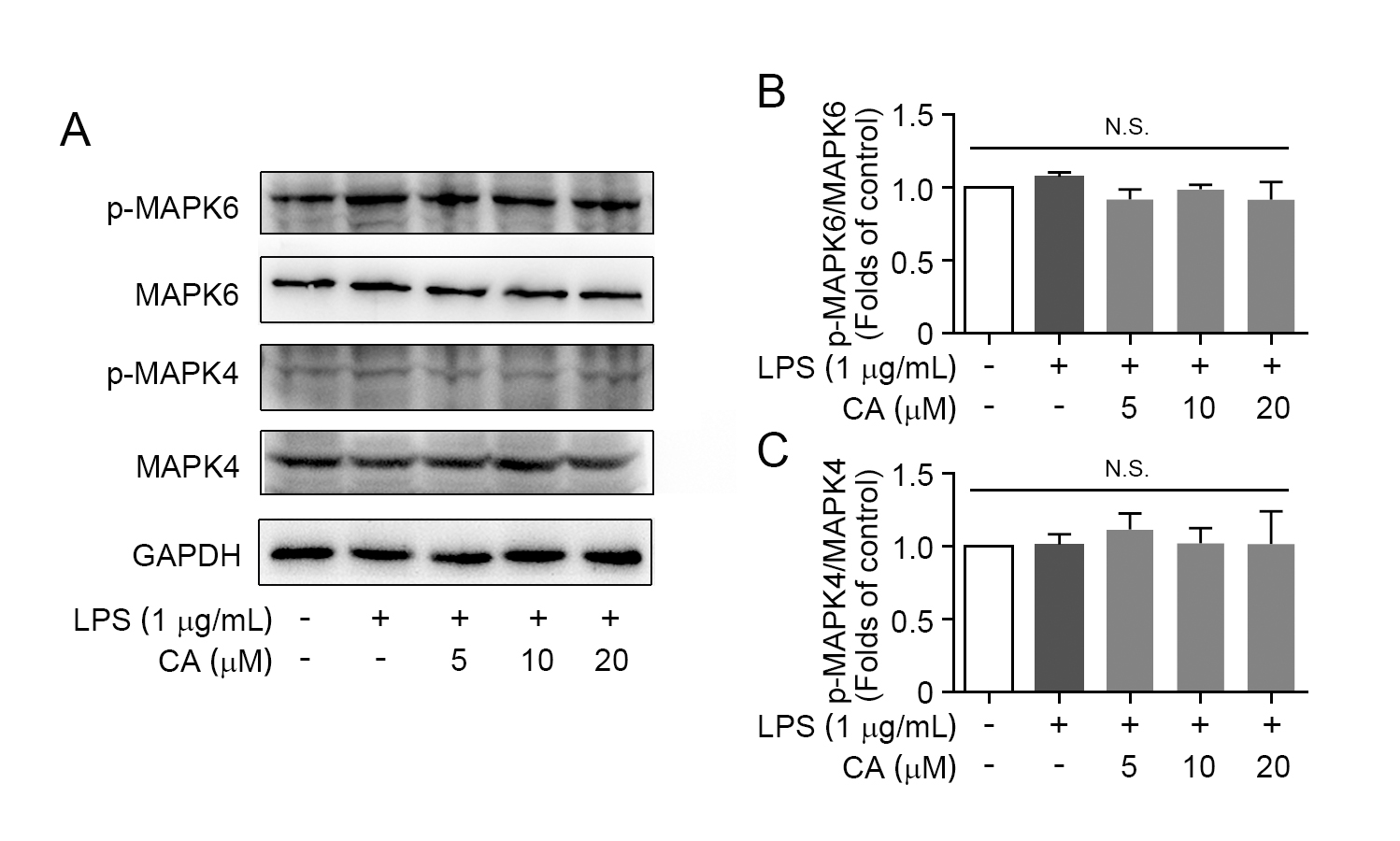
**

**Supplementary Figure 1** CA showed no significant changes on MAPK6 and MAPK4. Cells were treated with LPS (1 μg/mL) with or without CA (5, 10 and 20 μM) for 1 h. **(A)** Phosphorylation levels of MAPK6 and MAPK4 protein were determined by western blot assay. **(B-C)** Quantitative analysis for relative phosphorylation levels of MAPK6 (B) and MAPK4 (C) was performed by normalizing to the control group. Data are expressed as mean ± SEM for three individual experiments. N.S., not significant by ANOVA with Bonferroni’s *post-hoc* test.

**Supplementary Table 1. Primer pairs for real-time PCR**

| **Gene** | **Sequence** |
| --- | --- |
| ***Nos2*** | **F**: 5’- GCCCCTGGAAGTTTCTCTTCA-3’  **R:** 5’- CTGAGAACAGCACAAGGGGT-3’ |
| ***Tnfa*** | **F**: 5’-AAGCAAGCAGCCAACCAG-3’  **R:** 5’-CCACAAGCAGGAATGAGAAGA-3’ |
| ***Cox2*** | **F**: 5’-GGTGCCTGGTCTGATGATGTAT-3’  **R:** 5’-CCTATGAGTATGAGTCTGCTGGTT-3’ |
| ***Mcp-1*** | **F**: 5’-CTTCTGGGCCTGCTGTTCACAGTT-3’  **R:** 5’-TTCTTGGGGTCAGCACAGACCTCT-3’ |
| ***Foxo1*** | **F**: 5’-CCTTCTGGATAATCTCAACCT-3’  **F**: 5’-TGCTGTCCTGAAGTGTCT-3’ |
| ***Foxo3*** | **F**: 5’-GACGACCTGCTGGATAAC-3’  **R:** 5’-GTGACACGGAAGAGAAGG-3’ |
| ***Gapdh*** | **F:** 5’-GGTGAAGGTCGGTGTGAACG-3’  **R:** 5’-CTCGCTCCTGGAAGATGGTG-3’ |

**Supplementary Table 2. Pathway enrichment analysis for the CA-downregulated proteins**

| **No.** | **GO ID** | **GO Term** | **Ontology Source** | **Term P Value** | **% Associated Genes** | **Nr. Genes** | **Associated Genes Found** |
| --- | --- | --- | --- | --- | --- | --- | --- |
| **1** | GO:0000493 | MAPK signaling pathway | \| WikiPathway \| \| --- \| | 18.0E-3 | 3.14 | 5.00 | [Ecsit, Gck, Il1a, Il1b, Ptpn5] |
| **2** | GO:0001124 | MAPK6/MAPK4 signaling | REACTOME | 30.0E-3 | 2.11 | 8.00 | [Apc, Cdkn1a, Fzr1, Plk1, Ptch1, Rpgrip1l, Syngap1, Tnks] |
| **3** | GO:0004068 | FoxO signaling pathway | KEGG | 38.0E-3 | 3.03 | 4.00 | [Cdkn1a, Il6, Plk1, Rbl2] |
| **4** | GO:0000001 | Apoptosis | REACTOME | 42.0E-3 | 2.10 | 7.00 | [Apc, Cadm1, Cdh5, Col4a1, Col6a5, Jup, Mpp5] |
| **5** | GO:0005166 | HTLV-I infection | KEGG | 19.0E-3 | 2.47 | 7.00 | [Apc, Atf3, Cdkn1a, Il6, Nrp1, Pole3, Pole4] |
| **6** | GO:0005152 | Tuberculosis | KEGG | 28.0E-3 | 2.81 | 5.00 | [Cyp27b1, Il1a, Il1b, Il6, Ripk2] |
| **7** | GO:0001184 | Complex I biogenesis | \| REACTOME \| \| --- \| | 2.4E-3 | 6.78 | 4.00 | [Ecsit, ND4, Ndufaf3, Ndufs8] |
| **8** | GO:0000745 | CD28 co-stimulation | REACTOME | 2.9E-3 | 6.45 | 4.00 | [Cdh5, Jup, Nrp1, Plxna1] |
| **9** | GO:0000211 | Metabolism of nucleotides | REACTOME | 2.3E-3 | 5.21 | 5.00 | [Dctd, Dctpp1, Gda, Nme4, Nudt13] |
| **10** | GO:0000240 | Pyrimidine metabolism | KEGG | 3.2E-3 | 4.81 | 5.00 | [Dctd, Dctpp1, Nme4, Pole3, Pole4] |
| **11** | GO:0004360 | Axon guidance | KEGG | 27.0E-3 | 2.86 | 5.00 | [Nrp1, Plxna1, Ptch1, Ssh2, Ssh3] |
| **12** | GO:0001155 | Respiratory electron transport | REACTOME | 14.0E-3 | 4.08 | 4.00 | [Ecsit, ND4, Ndufaf3, Ndufs8] |
| **13** | GO:0004933 | AGE-RAGE signaling pathway in diabetic complications | KEGG | 15.0E-3 | 4.00 | 4.00 | [Col4a1, Il1a, Il1b, Il6] |
| **14** | GO:0004110 | Cell cycle | KEGG | 31.0E-3 | 3.23 | 4.00 | [Cdkn1a, Fzr1, Plk1, Rbl2] |

**Supplementary Table 3. Enrichment analysis of GO Biological process for the CA-downregulated proteins**

| **No.** | **GO ID** | **GO Term** | **Term P Value** | **% Associated Genes** | **Nr. Genes** | **Associated Genes Found** |
| --- | --- | --- | --- | --- | --- | --- |
| **1** | GO:0043123 | Positive regulation of I-κB kinase/NF-κB signaling | 1.7E-3 | 3.14 | 5.00 | [Ddrgk1, Fkbp1a, Il1b, Lgals1, Ripk2] |
| **2** | GO:0051092 | Positive regulation of NF-κB transcription factor activity | 3.4E-3 | 3.51 | 4.00 | [Ddrgk1, Il1b, Ripk2, Trim14] |
| **3** | GO:0043408 | Regulation of MAPK cascade | 28.0E-3 | 1.20 | 8.00 | [Apc, Atf3, Il1b, Il6, Nrp1, Psen1, Ripk2, Syngap1] |
| **4** | GO:0070371 | ERK1 and ERK2 cascade | 17.0E-3 | 1.81 | 5.00 | [Atf3, Il1b, Il6, Nrp1, Ripk2] |
| **5** | GO:0070555 | Response to interleukin-1 | 4.2E-3 | 3.31 | 4.00 | [Il1a, Il6, Ripk2, Taf9] |
| **6** | GO:0032722 | Positive regulation of chemokine production | 8.2E-6 | 9.80 | 5.00 | [Il1a, Il1b, Il6, Ripk2, Selenok] |
| **7** | GO:0050715 | positive regulation of cytokine secretion | 510.0E-6 | 4.13 | 5.00 | [Cadm1, Il1a, Il1b, Ripk2, Selenok] |
| **8** | GO:0019221 | cytokine-mediated signaling pathway | 6.2E-3 | 1.75 | 7.00 | [Ebi3, Fkbp1a, Ifitm3, Il1a, Il1b, Il6, Ripk2] |
| **9** | GO:1900180 | regulation of protein localization to nucleus | 6.6E-3 | 2.30 | 5.00 | [Cdkn1a, Il1b, Il6, Jup, Psen1] |
| **10** | GO:0002526 | acute inflammatory response | 5.2E-3 | 3.13 | 4.00 | [Il1a, Il1b, Il6, Serpina1a] |
| **11** | GO:0002682 | regulation of immune system process | 4.5E-3 | 1.17 | 15.00 | [Apc, C1rb, Cadm1, Cdkn1a, Fkbp1a, Gab2, Ifi202b, Il1a, Il1b, Il6, Lgals1, Psen1, Ripk2, Selenok, Sppl2a] |
| **12** | GO:0002263 | cell activation involved in immune response | 10.0E-3 | 2.04 | 5.00 | [Gab2, Il6, Lgals1, Psen1, Ripk2] |
| **13** | GO:0006915 | apoptotic process | 4.80E-02 | 1.27 | 23.00 | [Apc, Atf3, Cadm1, Cdkn1a, Cyp27b1, Ddrgk1, Degs1, Grid2, Ifi202b, Il1a, Il1b, Il6, Lgals1, Nme4, Nrp1, Plk1, Psen1, Ripk2, Selenok, Serpinb2, Syngap1, Taf9, Zfp830] |
